# Classifying EEG Signals during Stereoscopic Visualization to Estimate Visual Comfort

**DOI:** 10.1155/2016/2758103

**Published:** 2015-12-24

**Authors:** Jérémy Frey, Aurélien Appriou, Fabien Lotte, Martin Hachet

**Affiliations:** ^1^Université de Bordeaux, Potioc Project-Team, 351 Cours de la Libération CS 10004, 33405 Talence Cedex, France; ^2^Inria, Inria Bordeaux Sud-Ouest, Potioc Project-Team, 200 Avenue de la Vieille Tour, 33405 Talence Cedex, France

## Abstract

With stereoscopic displays a sensation of depth that is too strong could impede visual comfort and may result in fatigue or pain. We used Electroencephalography (EEG) to develop a novel brain-computer interface that monitors users' states in order to reduce visual strain. We present the first system that discriminates comfortable conditions from uncomfortable ones during stereoscopic vision using EEG. In particular, we show that either changes in event-related potentials' (ERPs) amplitudes or changes in EEG oscillations power following stereoscopic objects presentation can be used to estimate visual comfort. Our system reacts within 1 s to depth variations, achieving 63% accuracy on average (up to 76%) and 74% on average when 7 consecutive variations are measured (up to 93%). Performances are stable (≈62.5%) when a simplified signal processing is used to simulate online analyses or when the number of EEG channels is lessened. This study could lead to adaptive systems that automatically suit stereoscopic displays to users and viewing conditions. For example, it could be possible to match the stereoscopic effect with users' state by modifying the overlap of left and right images according to the classifier output.

## 1. Introduction

Stereoscopic displays have been developed and used for years in computer science, for example, to improve data visualization [1, 2], to ease collaboration between operators [3], or to better manipulate virtual objects [4]. However, it is only during the past decade that this technology began to reach users beyond experts. Notably, movie theaters, and the entertainment industry in general, helped to popularize the so-called “3D” contents. Nowadays stereoscopic displays are used at home. “3D” television sets gain in popularity and game devices started to use this technology. Yet, whenever devices use shutter or polarized glasses, parallax barrier (e.g., Nintendo 3DS) or head-mounted displays (as with the Oculus Rift) to produce pairs of images, visual discomfort could occur when the stereoscopic effect is too strong. Some viewers could even suffer pain [5].

In order to mitigate those symptoms and adapt the viewing experience to each user, we propose an innovative method that can discriminate uncomfortable situations from comfortable ones. It reacts quickly (within 1 s), without calling upon users, so it does not disrupt the viewing.

Our solution is versatile because all stereoscopic displays use the same mechanism to give the illusion of depth. They send a different image to the left and right eyes. As with natural vision, the visual fields of our eyes overlap and the difference between the two images helps our brain to estimate objects' distance.

To facilitate images' merge, observers rely on two mechanisms. First, they need to maintain the point of interest at the same place on their both retinas. This is why the closer an object gets, the more eyeballs rotate inward. This is called “vergence,” and it also happens with stereoscopic displays. Second, in a way similar to how camera lenses operate, crystalline lenses need to focus light beams. They deform accordingly to objects' position in order to obtain a clear picture. This other physiological phenomenon is called “accommodation” and is* not* replicated with stereoscopic displays.

**Figure 1:**
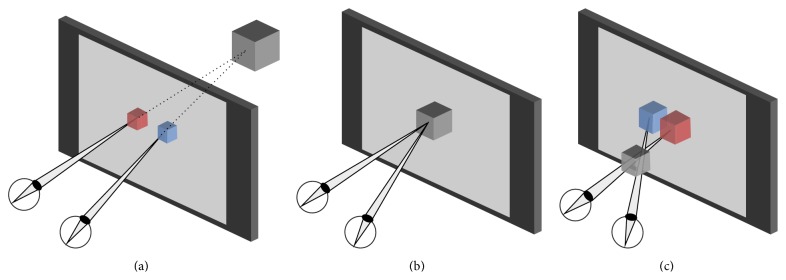
The vergence-accommodation conflict (VAC). (a) Object “behind” the screen, negative VAC. (b) The object appears flat, no VAC. (c) Object “in front,” positive VAC.

In a natural environment, vergence and accommodation are locked to objects' positions and occur altogether. However, since the focal plane in stereoscopic displays is fixed, accommodation will not change. No matter how far or how close virtual objects appear to be, physical screens remain at the same place. The discrepancy between vergence and accommodation is called the “vergence-accommodation conflict” (VAC, see Figure 1). It causes stress on users [5]. The closer or further a virtual object gets compared to the display plane, the stronger this conflict is. When it is too important or lasts too long, visual discomfort occurs.

**Figure 2:**
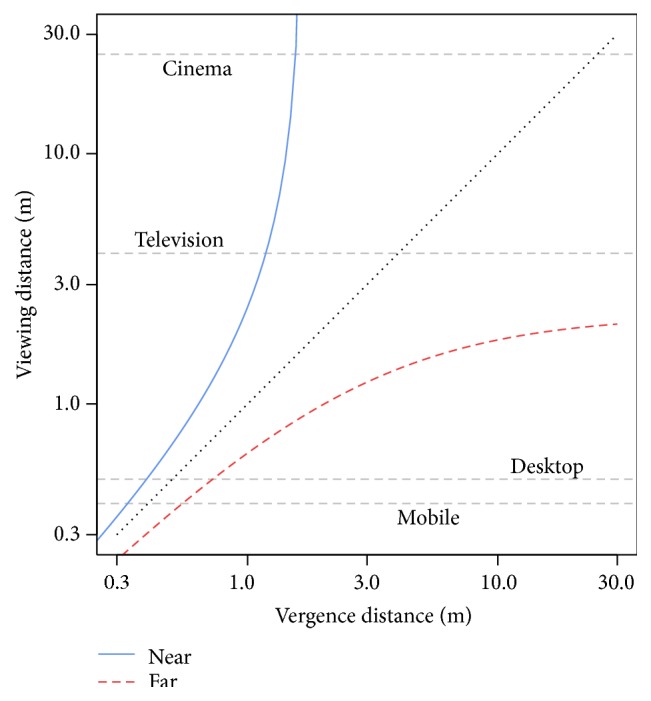
The acceptable zone of comfort depending on viewing distance and vergence distance, that is, the apparent depth of contents. From [7].

VAC is one of the major causes of the symptoms associated with visual discomfort and visual fatigue in stereoscopic displays [5, 6]. Guidelines exist to limit the VAC and prevent such negative effects. In particular, Shibata et al. [7] established a “zone of comfort” using questionnaires, a zone within which the apparent depth of objects should remain to avoid discomfort (see Figure 2). It takes into account the distance between viewers and displays. Unfortunately, individual differences [5] make it hard to generalize such recommendations and use them as is. Besides, viewing conditions vary. Ambient light, screen settings, viewing angle, and stereoscopic techniques are parameters among others that influence the rendering and as such alter visual strain [8].

New investigation techniques record users' physiology. Complementary to qualitative questionnaires, as used in [7], brain activity recordings enable the monitoring of users' states [9–11]. One of the main advantages of such a technology for the evaluation of human-computer interaction (HCI) comes from the real-time insights that it could give. In [12], the authors demonstrate with functional magnetic brain resonance imaging (fMRI) how stereoscopy increases intersubject correlation of several neural networks, overlapping data with the time course of a movie, and how it reflects immersive tendencies reported via questionnaires.

Electroencephalography (EEG) is among the cheapest and most lightweight devices that sense brain signals. Even though EEG has been used to investigate visual fatigue induced by stereoscopic display [13–16], those studies only compared flat images with stereoscopy. They do not control for objects virtual positions; hence they cannot account for different comfort conditions. Furthermore, most of the EEG studies related to stereoscopic display and comfort analyzed stimuli which last several minutes (e.g., from 3 to 40 min in [13, 15, 16]). Such protocols could not lead to adaptive systems that react quickly; they focus more on the overall fatigue induced by a prolonged exposition to stereoscopy rather than discomfort. Some other works have measured in EEG signals the perceived quality of 2D videos [17, 18]. While being interesting and relevant, such work only used 2D videos and thus did not address stereoscopic comfort.

In this paper, we propose and validate a system (note that we recently presented preliminary evaluations of such a system in a conference short paper [19]) that classifies EEG signals to measure visual comfort on short time windows (few seconds). This type of system is a brain-computer interface (BCI [11]). Unlike most other user interfaces, BCIs do not rely on muscle activity, but only on brain signals. Because our system does not use brain patterns that are produced deliberately by users, such as the imagination of hand movements, it is called a* passive* BCI [10, 20]. More and more passive BCIs are used to monitor users' state in order to improve HCI. For example, it is possible to monitor in real-time the workload of a person [21, 22], the amount of cognitive resources that are allocated, and to adapt consequently the difficulty of the task on-the-fly [23]. Such type of application, namely, adaptive systems increasing both users' comfort and performance, drove our research.

Our main contribution is to prove the feasibility of an EEG system that could estimate in near real-time (1 s delay) the visual comfort viewers are experiencing as they watch stereoscopic displays. It could be adapted to real-case scenarios by controlling the discrepancy between left and right images depending on the output of the classifier. Then it could be employed in different settings to ease users' comfort, for example, when they manipulate 3D contents during prolonged periods of time, such as remote design or video games, or when people are watching 3D movies, especially when there are many relatively rapid depth variations, as seen in action sequences.

## 2. Experiment

### 2.1. Overview

**Figure 3:**
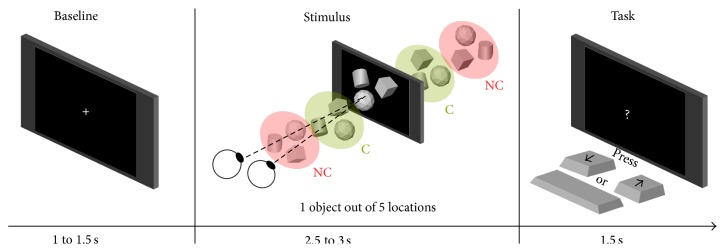
One trial: cross (baseline), object at random depth, task.

We studied the appearance of virtual objects. They were presented to participants at different apparent depths for a few seconds (see Figure 3). We created two conditions: objects appeared either at a Comfortable position (“C” condition) or at a position that is Not Comfortable (“NC” condition).

We displayed simple grey objects over a black background. Three kinds of primitives were employed: cube, cylinder (32 vertices), and icosphere (80 faces); curves and surfaces' size are important for objects' comprehension [24]. Objects' orientations were randomized along the three axes to create various stimuli [25]. Rotations were controlled so as the faces of cubes and cylinders could not be orthogonal to the camera plane, thus preventing the appearance of artificial 2D shapes. The resulting 3D scenes were kept simple enough to ensure that there were no distracting elements and that no variables beside the VAC were manipulated. We deprived the depth cues to control for VAC. For example, casting shadows would have helped to differentiate close objects from far objects without the need of binocular fusion [26].

We defined ranges inside and outside the zone of comfort based on the results from [7]. In [7], the ranges for the comfortable and uncomfortable zones were determined experimentally and on average over participants, based on a questionnaire administered to multiple participants after long (45 minutes) stereoscopic visualizations. We selected ranges that were clearly in the comfortable/uncomfortable zones, and not at the boundaries between zones, to ensure that the displays were really comfortable/uncomfortable for all participants. Related to the location of participants sitting 1 m away from the display, in “C” condition virtual objects were positioned within [0.75 m; 0.85 m] (comfortable close) or within [1.3 m; 1.6 m] (comfortable far). In “NC” conditions, ranges were [0.35 m; 0.45 m] (uncomfortable close) or [4 m; 6 m] (uncomfortable far). During one-third of the trials, objects appeared “flat” (no stereoscopic effect, 1 m apparent depth, as far as the screen).

In order to assess their capacity to situate virtual objects in space and to maintain their vigilance high during the whole experiment, participants had to perform a task. When a question mark was shown on screen, “down” arrow, “space” bar, or “up” arrow were pressed to indicate whether objects appeared “in front of,” “as far as” (flat images), or “behind” the screen. With both hands on the keyboard, choosing those keys to answer ensured that participants' gaze was not leaving the screen and that participants movements would not pollute EEG signals.

A trial started with a neutral stimulus, a 2D cross appearing on screen for a duration comprised between 1 and 1.5 s. Then the virtual object appeared for 2.5 to 3 s. Finally, a question mark appeared for 1.5 s, a period during which participants had to perform the task. After that, a new trial began. This sequence is illustrated in Figure 3. The first two time intervals, that randomly varied by 0.5 s, prevented participants from anticipating objects appearance and the moment they had to respond to the task. On average a trial took 5.5 s. All in all there were 160 trials per C and NC conditions. Trials were equally split across 4 subsessions to let participants rest during the investigation and avoid a too tedious experiment.

### 2.2. Apparatus

Stereoscopic images were shown in full HD resolution (1080 p) on a 65-inch Panasonic TX-P65VT20E, an active display; participants wore shuttered glasses. The software that rendered the virtual objects was programmed with Processing framework, version 2.2.1. Objects were dynamically created. No matter their apparent depths, primitives sizes on screen remained identical: they were scaled within the virtual scene. In combination with a diffuse illumination of the scene, this made it impossible to discriminate conditions without stereoscopy. The interpupillary distance used to compose stereoscopic images was set at 6 cm, an average value across population [27].

EEG signals were acquired at a 512 Hz sampling rate with 2 g.tec g.USBamp amplifiers (http://www.gtec.at/). This medical grade equipment handles 32 electrodes. We used 4 electrodes to record specifically electrooculographic (EOG) activity and 28 to record EEG. In the international 10–20 system, EOG electrodes were placed at LO1, LO2, IO1, and FP1 sites; EEG electrodes were placed at AF3, AF4, F7, F3, Fz, F4, F8, FC5, FC1, FC2, FC6, C3, Cz, C4, CP5, CP1, CP2, CP6, P7, P3, Pz, P4, P8, PO3, PO4, O1, Oz, and O2 sites. OpenViBE 0.17, a graphical user interface oriented toward EEG signal processing [28], recorded both electrodes' signals and the key strokes of the task.

**Figure 4:**
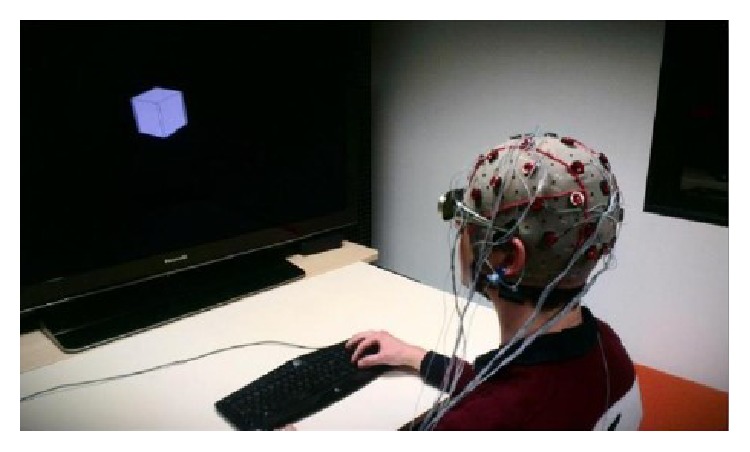
Setup of the experiment, with a subject being presented with stereoscopic images while his EEG signals are being recorded.

OpenViBE was also used to trigger images appearance. To do so, TCP messages were sent from OpenViBE to Processing. The same machine ran both programs; thus TCP latency was negligible (<1 ms). 3D rendering on Processing side could necessitate some CPU cycles though, and event-related potentials (ERP) analyses suffer from bad synchronizations. This is why we took extra precautions to accommodate rendering delays and ensure a reliable synchronization between objects' appearance and EEG recordings. Processing framerate was reduced down to 25 FPS and a 60 ms interval was set between TCP messages interception and the appearance of a new image, a sufficient time for the machine to make the virtual rendering and avoid lags. Overall, this mechanism ensured a constant 100 ms delay between sent messages and images appearance. The whole setup can be seen in Figure 4.

### 2.3. Participants

12 participants took part in the experiment: 5 females, 7 males; mean age 22.33 (SD = 1.15). They reported little use of stereoscopic displays: 1.91 (SD = 0.54) on a 5-point Likert scale (1: never; 2, 3, 4, and 5: several times a year/month/week/day, resp.). If applicable, participants wore their optical corrections and there was enough space beneath the shutter glasses for regular glasses not to disrupt user experience.

We made sure that no participant suffered from stereo blindness by using a TNO test [29]. We created a computerized version of this test to ensure that their ability to perceive stereoscopic images was on par with our equipment, as advised in [30].

### 2.4. Measures

Beside EEG measures, task scores were computed from participants' assessment of objects' virtual position in space, whether they appeared “in front of,” “as far as,” or “behind” the screen. During the 1.5 s time window when question marks appeared, the first key pressed, if any, was taken into account. A correct answer resulted in 1 point, an incorrect in −1 point, and none in 0 points. Final scores were normalized from [−480; 480] (3*∗*160 trials for C, NC, and 2D displays) to [−1; 1] intervals.

A questionnaire inquiring the symptoms associated with the different apparent depths preceded first trials and followed each subsession. There were 2 items, one asking about participants' vision clarity and the other about eyes tiredness. The corresponding 5-point Likert scales were adapted from [7], “1” representing no negative symptoms and “5” severe symptoms. We measured, respectively, how well participants saw the stereoscopic images and how comfortable they felt; to do so we averaged the answers (10 values per item and per C/NC conditions).

### 2.5. Procedure

The experiment occurred in a quiet environment, isolated from the outside, with a dimmed ambient light. The whole experiment was approximately 90 minutes long and comprised the following steps:

1. Participants entered the room. They were seated 1 m away from the stereoscopic screen (distance from their eyes), next to a table. They read and signed an informed consent form and filled a demographic questionnaire.
2. The stereoscopic display was switched on and participants' stereoscopic vision was assessed with a TNO test.
3. An EEG cap was installed onto participants' heads and we ensured reliable EEG signals recordings.
4. The “symptoms” questionnaire was given orally, experimenter manually triggering objects appearances. There was 1 object per virtual depth range (C close/far, NC close/far) and 2 flat objects, making 6 randomized objects per questionnaire.
5. A training session occurred. During this session participants had the opportunity to get familiar with the trials and with the task. We waited until participants felt confident enough and were ready to proceed with the main part of the experiment.
6. The 4 subsessions, described previously, occurred. When a subsession ended, participants were given again the questionnaire of step 4 before they could rest, drink, and eat. Once they felt ready, we pursued with the next subsession.

## 3. Analyses

Because we want to increase fundamental knowledge on brain activity, we were particularly cautious to base our analyses on “clean” EEG signals, that is to say, on EEG signals not polluted by artifacts such as eye movements [31]. The signal processing that we present in this section uses state-of-the-art tools to remove such artifacts. In Section 4 we will explain how the use of a simplified pipeline, one that could be easily applied online in real-life scenarios, has little impact on performance.

### 3.1. EEG Signal Processing

We used EEGLAB 13.3.2b [32] and MATLAB R2014a to process EEG signals offline. Data gathered from the 4 subsessions were concatenated. We applied a 0.5 Hz high-pass filter to correct DC drift and a 25 Hz low-pass filter to remove from our study signal frequencies that were more likely to be polluted by muscle activity. We extracted the 320 epochs of EEG signals around C and NC stimuli onsets, from −1 s to +2.5 s.

Due to the important amount of data (3840 trials across our 12 participants), we chose automated methods to clean the signals. The EEGLAB function pop_autorej removed epochs that contained muscular artifacts. Following the results obtained in [33], EOG activity was suppressed from the signal using the ADJUST toolbox 1.1 [34]. After an Infomax independent component analysis (ICA), we rejected components that ADJUST labeled as eye blinks or eye movements (vertical and horizontal).

**Figure 5:**
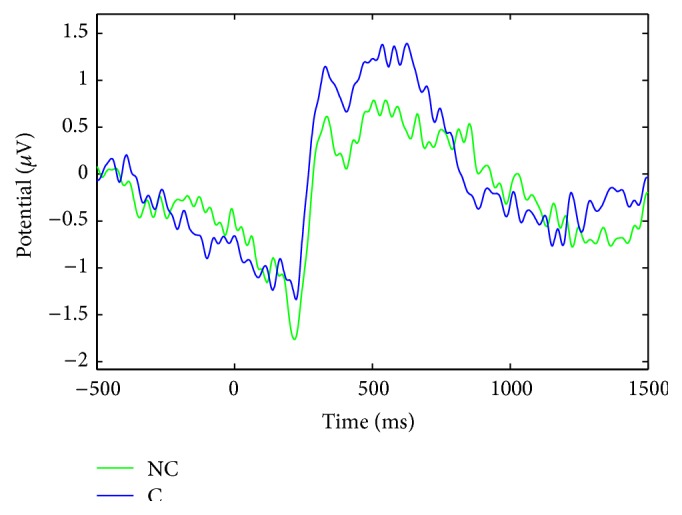
Average ERP across 28 EEG electrodes and 12 participants. Blue: comfort condition; green: no-comfort condition (≈160 trials each). The stereoscopic object appears at *t* = 0 ms.

We analyzed the event-related potential (ERP) following the appearance of stereoscopic images. Averaged ERPs across participants indicated that ERPs had a higher positive peak in C (see Figure 5).

**Figure 6:**
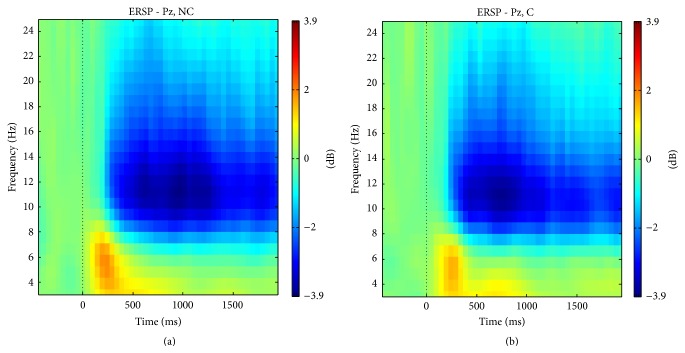
Average ERSP in Pz (medial parietal region). (a) No-comfort condition; (b) comfort condition (≈160 trials each, 12 participants).

There were some differences in EEG oscillations, event-related spectral perturbations (ERSP), depicted in Figure 6. Overall, there may be notably both a decrease of signal power within the alpha band (≈7 Hz–13 Hz) and an increase within the theta band (≈4 Hz–6 Hz) in no-comfort condition. Based on these findings over averaged trials, we employed spectral domain information with different features extraction methods and different classifiers for single trial classification. The benefits derived from the combination of temporal (ERP) and spectral (band power) characteristics were minor compared to the growing complexity of the underlying signal processing. For the sake of the argument, we preferred to detail a more intelligible framework in this section and to relegate a brief description of the combination of features in Section 4. This is why our classification strategy solely relies on temporal information when we compared different pipelines, for example, Monte Carlo simulations, pseudoonline and reduced number of electrodes (see below).

### 3.2. Classification

We used a common pipeline to classify EEG signals. Basically, it consists in extracting relevant signal features, training the classifier on a certain set of data, it corresponds to a “calibration” phase, and then testing the classifier performances on unseen data, which simulates a real-case application.

We split the EEG dataset of each participant into two. The first half of the trials was used as a training set and the second half as a testing set. This distribution facilitates the comparison between offline and online signal processing. In order to utilize temporal information, feature extraction relied on regularized Eigen Fisher spatial filters (REFSF) method [35]. This spatial filter, specifically designed for ERPs classification, reduced signals dimension from 28 EEG channels to 5 “virtual” channels whose signal is more discriminant between conditions. Note that we did not include in our study the 4 channels that were specifically recording EOG activity.

We selected a time window of 1 s, starting at *t* = 100 ms to accommodate the fixed delay with objects appearances (see Section 2.2). In order to reduce the number of features, we decimated the signal by a factor 16. As a result, there was 160 features by epoch (5 channels × 512 Hz × 1 s/16). We used shrinkage LDA (linear discriminant analysis) as a classifier [36]. Shrinkage LDA algorithm is more efficient compared to regular LDA when it comes to a high number of features [37].

### 3.3. Simulating Longer Stimuli with Monte Carlo

Although we used 1 s time windows as a basis for our analyses, we wanted to go beyond and test longer stimuli by aggregating trials.

We could not use directly the data we gathered because in our experimental protocol conditions were randomized. So we had to simulate. We used Monte Carlo simulations to cluster trials. The principle is as follows: studying 3 presentations, we cluster 3 similar trials drawn from the testing set (e.g., “no-comfort”, 3 × NC). Then we look at individual classifications from the system (e.g., NC–NC–C) and keep the label which has the majority; in the case NC, the resulting classification is correct for this cluster. Had the classifier labelled trials as “C–C–C,” “NC–C–C,” “C–NC–C,” or “C–C–NC,” the cluster would have been erroneously labeled as “C.”

Different combinations of trials were drawn from the testing set to compute the scores for *n* = 3, 5, and 7. Monte Carlo simulations served two purposes. On the one hand, it simulates the behavior of the classifier over a longer sequence of identical stimuli. On the other hand, and reciprocally, it allows the experimenter to suit the stimuli to the performance she or he wants to obtain for the desired use-case. Indeed, with an “*n*” as big as one wants, the trade-off between accuracy and exposure time could be freely chosen.

## 4. Results

### 4.1. Task and Symptoms Questionnaires

We used a Wilcoxon signed-rank test to compare task scores between C and NC conditions (means: 0.45* versus* 0.40). There was no significant effect (*p* = 0.78).

A Wilcoxon signed-rank test showed a significant effect of the C/NC conditions on both symptoms items (*p* < 0.01). Participants reported more eye comfort (means: 2.41* versus* 3.46) and more vision clarity (means: 2.10* versus* 3.13) in C than in NC.

### 4.2. Classification

We were able to predict with an average classification accuracy of 63.30% (SD = 7.64) the visual comfort experienced by viewers (see Table 1). We studied further this first result on 3 different aspects: we used Monte Carlo simulations to improve performances over longer stimuli; we investigated how the classifier behaves when only half of the EEG electrodes are employed; and finally we simulated an online analysis to assess performance in a real-life scenario. Those results are detailed below and summarized in Table 2.

#### 4.2.1. Monte Carlo Simulations

**Figure 7:**
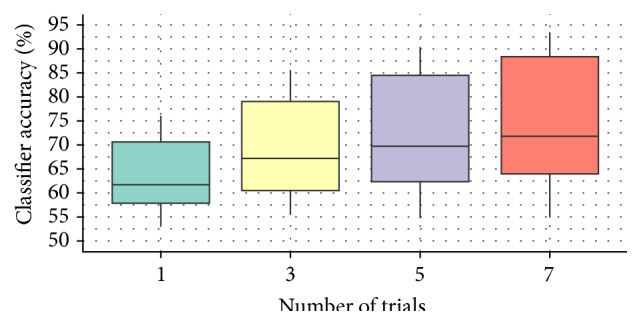
Monte Carlo simulations, classifier accuracy depending on the size of trials' clusters.

With Monte Carlo simulations, we investigated how the system would perform with the appearance of several images from the same condition. Classifier accuracy reached 68.91% (SD = 10.32) over 3 trials. Over 5 trials the classification reached 90% for some users, resulting in a 71.83% average (SD = 12.28). With *n* = 7, one-third of the participants reached 90% or more (74.08% on average, SD = 13.39). See Figure 7.

#### 4.2.2. Channels' Contribution: Accuracy over 14 Channels

EEG device that possesses fewer electrodes would be more comfortable to wear, faster to set up, that is, more practical, and less expensive.

**Figure 8:**
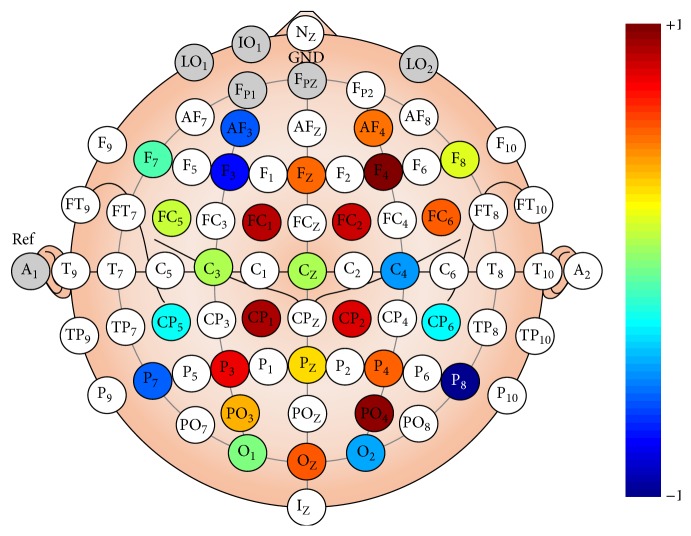
EEG channels' contribution to the spatial filter used by the classifier, averaged across participants. The unit of the scale is arbitrary, from “−1” (least important) to “+1” (most important).

We studied which channels contributed the most and which contributed the least to the classifier output. For each channel, we averaged across participants the absolute value of the spatial filter's coefficients that were computed by the REFSF extraction method. We arbitrarily normalized the data between −1 and 1 for more clarity (see Figure 8). This normalization to [−1; 1] was performed for each subject according to the minimum and maximum values of the spatial filters weights for this subject that were, respectively, mapped to −1 and 1.

To assess the performance of a BCI system that would use less EEG electrodes, we retained the upper half of the channels (i.e., 14 channels out of 28) that contributed the most to features' extraction using these computations (i.e., based on the average absolute channel weights in the spatial filters, as described above), that is, F4, PO4, CP1, FC1, FC2, CP2, P3, Oz, FC6, P4, Fz, AF4, PO3, and Pz. With the reduced set of 14 EEG channels, the classifier resulted in a 62.77% accuracy (SD = 7.47), which is close to the configuration that includes all channels.

#### 4.2.3. Online Scenario

The pipeline that we presented in Section 3 would be difficult to apply in real-life scenarios; online analyses prevent the use of advanced signal processing, such as ICA for artifact removal, because it requires heavy computations and often necessitates the entire EEG trace to be effective. Fortunately, artifacts had little incidence on the performance of our system. We simulated an online pipeline by skipping several steps—we removed ICA decomposition and did not use neither the ADJUST toolbox nor the pop_autorej function from EEGLAB—and still obtained similar results, with an accuracy of 62.40% (SD = 4.80).

#### 4.2.4. Combining with Frequency Bands

Interestingly enough, we managed to improve the performance of our system by combining temporal features (i.e., ERPs) with spectral features (“frequency bands”). Besides REFSF for temporal features, we used common spatiospectral patterns (CSSP) to extract spectral features [38]. Four frequency bands were extracted using a Butterworth filter with order 3: delta (1 Hz–3 Hz), theta (4 Hz–6 Hz), alpha (7 Hz–13 Hz), and beta (14 Hz–25 Hz). Concurring with ERSP analyses and the time course in Figure 6 that depicts differences between C and NC conditions, the best results were reached by extracting spectral features over a 1 s time window that started at *t* = 1100 ms (1000 ms + 100 ms for image appearance delay). REFSF and CSSP features were concatenated and normalized (*z*-score). Using a feature selection method based on the ratio of features' medians [39], we reduce the number of features passed to the classifier from 184 to 50; there were at the beginning 160 features from REFSF + 24 features from CSSP, 3 pairs from 4 bands. In the end we obtained a 64.66% accuracy (SD = 5.79). Note, however, that this 64.66% classification accuracy is not statistically different from the 63.30% accuracy obtained using only ERP (Wilcoxon test, *p* > 0.05). This therefore suggests that although the spectral features do contain relevant information for classification, this information might not be different from the one contained in ERP. Alternatively, maybe the approach we used to combine these two kinds of information was not optimal.

### 4.3. Factors Influencing Classification

We investigated which personal factors could influence the results of our classifier. Outside EEG recordings, the data that reflected most participants intervariability was concealed among the task's scores and the symptoms associated with stereoscopy. We used Spearman's rank correlation to test between, on the one hand, classifier accuracy and, on the other hand, the difference between NC/C scores and NC/C answers to symptoms questionnaires.

There was no significant association neither with the performance task (*p* = 0.44), with eye comfort (*p* = 0.81), nor with vision clarity (*p* = 0.57).

## 5. Discussion

During short exposures to images, participants reported worse vision clarity and less visual comfort in NC condition, thereby validating a clear distinction between the two zones of comfort of our protocol. Participants performed equally well in both conditions during the task, suggesting that, even if severe, a VAC does not alter their ability to make rough estimations of virtual depths. In this context, it also highlights the limits of behavioral methods in measuring participants' comfort. A neuroimaging technique, on the other hand, did manage to discriminate two comfort conditions.

EEG signals reflected the disparities in visual comfort. We mainly focus our computations on ERPs, as temporal features led to a signal processing pipeline that was both comprehensible and effective. Using an offline analysis, it was possible to build a classifier that achieved an accuracy greater than 63%, with several participants exceeding 70%. The system scored above chance level in all our analyses (*p* < 0.01) [40]. The performance of the classifier was not influenced by participants' ability to perceive depth nor by the strain that induced the presentation of stereoscopic images.

This score of 63% accuracy, while being not as high as some other established BCI systems, may be already sufficient to improve users' comfort. Indeed, on-the-fly correction of uncomfortable images can be seen as error correction, and in such settings detection rates from 65% are acceptable to improve interactions [41]. These findings depend on the nature of the task, of course. This is why we proposed a mechanism to increase the performance of the classifier.

By taking into account more than one object appearance, or by increasing the duration of viewing sessions, the classifier should become more reliable. The system score improved by 6 points when we clustered trials by 3. During our simulations, the accuracy went around 90% for some users with 5 trials and for one-third of the participants over 7 trials. It is possible to use this method to simulate an arbitrary number of consecutive trials. Therefore, this tool can estimate how many presentations are needed to reach a specific accuracy and suit the desired application.

**Figure 9:**
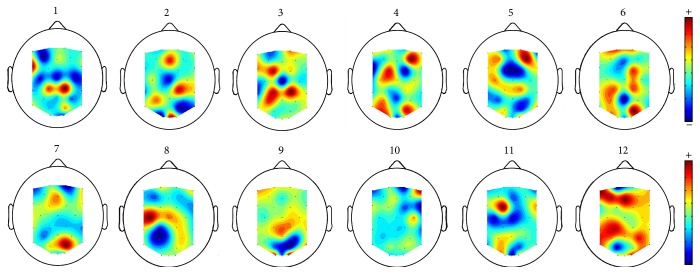
Top spatial filter (i.e., spatial filter corresponding to the largest eigenvalue in the REFSF algorithm) for each participant.

We observed a rather large interparticipant variability in terms of obtained classification accuracy. While we do not have a definitive explanation about such interparticipants variations, a number of hypotheses may explain these results such as differing levels of attention during the experiment (which was a bit boring) across subjects, different experience and habituation with stereoscopic displays and thus different cortical processing of such stimulus, different interpupillary distances, different levels of perception of stereoscopy, or the more usual differences in cortex organization and skull thickness that may change the resulting EEG signals and ERP amplitude. In addition, we also visualized the top spatial filter for each participant (i.e., spatial filter corresponding to the largest eigenvalue in the REFSF algorithm) to look for alternative explanations. These spatial filters can be seen in Figure 9. It appeared that the “best” participants (accuracy > 70%, i.e., participants 4, 8, 9, and 10) had spatial filters with more focally located large weights, mostly in frontal and occipital areas, whereas the “worst” participants (accuracy < 60%, i.e., participants 1, 2, 3, 11, and 12) had large channel weights more spread over the whole cortex, which may suggest the presence of more noise or task-irrelevant signals. Note that this might also be due to differences in attention as mentioned above.

During our study we found differences among frequency bands power. While those differences were not large, they might present an opportunity to strengthen classifier's performance. While in our experiment combining temporal and spectral features did not significantly improve classification accuracy, further investigations that explore other combination schemes should be performed in the future.

We were able to replicate our results with a simplified pipeline that could be applied online, paving the way for real-life applications. Furthermore, we were able to select the EEG channels that contributed the most to classifier performance and to halve their number with little loss in accuracy. Even though we used a medical grade EEG equipment to set the basis of a new adaptive system, it seems to indicate that our system could remain functional with entry-level devices. As a matter of fact, the reduced number of channels that we used, 14, correspond to the number of EEG electrodes found on the Emotiv EPOC (https://emotiv.com/). With the EPOC the positioning of the electrodes is constrained by the manufacturer, but other initiatives, such as the 16-channel OpenBCI system (http://www.openbci.com/), may combine affordability, flexibility, reliability, and ease of use.

## 6. Conclusion

We described an innovative system that can distinguish uncomfortable stereoscopic viewing conditions from comfortable ones by relying on EEG signals. We controlled the experimental conditions with questionnaires, founding significant differences in visual comfort between short exposures of images. Visual* comfort* was assessed, whereas existing studies focused on visual* fatigue*, a component that appears in the long term and that we propose to prevent beforehand.

A passive stereoscopic comfort detector could potentially be useful for multiple applications, as a tool to (1) objectively compare (possibly offline) different stereoscopic displays, (2) dynamically enhance stereoscopic effects, by increasing discrepancy without causing discomfort, (3) quickly calibrate stereoscopic displays, and (4) dynamically adapt discrepancy to avoid discomfort (e.g., during 3D movies) or voluntarily cause discomfort (e.g., for basic science studies about perception), among many others.

Using short time windows (features were extracted over 1 s), we set the basics of a tool capable of monitoring user experience with stereoscopic displays in near real-time. Our offline analysis used the state of the art in signal processing to demonstrate the feasibility of such a method with clean EEG signals. We obtained a similar classification accuracy without computationally demanding artifacts filtering, demonstrating also that the work presented here could perfectly be applied online. A framework like OpenViBE could ease the creation of an online scenario. Even though some BCI applications are biased by artifacts not originating from brain activity, for example, emotion recognition by facial expression [42], during our investigations we discovered that eye artifacts did nothing but adding slight noise to the system. Either an automatic removal method could be employed to clean the signal online [43] or the EEG electrodes could be positioned over the parietal and occipital regions.

More complex signal processing can increase classification rate. We also described a method that can assess how many stimuli are needed to reach a particular accuracy, that is, Monte Carlo simulations.

Although it is not deniable that it is currently easier to calibrate displays without EEG, a passive BCI can adapt the parameters to users' state throughout the viewing. It is complementary to other methods that aimed at improving users' comfort. It is possible to integrate EEG measures with other physiological sensors, as hinted by other systems [16].

At the same time that a passive BCI that could adapt viewing conditions to users is built, experimental protocol should be enhanced to integrate richer stimuli. Colors, shadows, relative positions, or movements are different cues that can participate in the comprehension of depth. Besides, real-life scenarios involve virtual scenes that are more complex than grey images of primitive shapes. It will also lead to a broader VAC spectrum. Even if the “curse-of-dimensionality” [44] will prevent a classifier from possessing many classes, the more VAC are taken into account, the more refined adaptive systems will be.

In order to make the adaptive system reliable and useful for the many, differences between individuals that influence classifier performance need to be studied. Physiological characteristics, for example, interpupillary distance, and past experience with stereoscopic displays—some people may need more time to acclimate to such technology—should be considered. Users' states also have to be taken into account; for example, mental fatigue likely relates to visual fatigue. This study will hereby lead to promising work in many fields: human factors to understand brain patterns disparities, signal processing to improve accuracy, design to create adaptive interfaces, entertainment to integrate comfort measures, and manufacturers to create more accessible hardware solutions and popularize the use of EEG. By combining those different areas of expertise, passive BCIs should become a viable option for increasing users' comfort, a solution that does not disrupt work or the narrative.

The transition toward more practical settings should be seamless, as classifier performance remains stable even when half the EEG electrodes are used. Next step would consist in conceiving an analogous online system that monitors more complex virtual scenes. A real-world application could consist in a gamified version of our task that smoothly corrects depth range upon classifier output. Such smooth alteration could be applied to animation movies as well. The discrepancy between left and right images would be gradually reduced while discomfort is detected, for example, when several presentations of objects that are virtually close to the users trigger such label within the classifier. On the contrary, the discrepancy could be increased gradually to enhance the stereoscopic effect as long as no discomfort is detected. This requires only the tuning of one parameter of the display, which is accessible, for example, through devices such as the Nintendo 3DS or the Nvidia 3D vision system. When the content is dynamically generated, that is, video games, the control over the virtual scene is even more substantial. In this case one could differently adapt objects' position, according to whether they seem to appear in front of the screen or behind it.

We documented a novel solution to a famous issue, that is, estimating stereoscopic discomfort, thus increasing fundamental knowledge. Besides 3D scenes control, by giving access in real-time to users' inner states, EEG will help to modulate more closely the viewing experience according to the effect one wants to achieve.

## Figures and Tables

**Table 1:** Classifier accuracy (in percentage) for every participant. Mean: 63.30%, SD: 7.64.

Participant	1	2	3	4	5	6	7	8	9	10	11	12
Accuracy	54.17	59.23	58.22	70.32	60.53	64.19	62.91	76.06	72.46	71.52	53.24	56.74

**Table 2:** Overview of the classifier performance for the various methods we investigated.

Method	Accuracy	SD
Offline pipeline (ERP)	63.30%	7.64

Monte Carlo (ERP, *n* = 3)	68.91%	10.32
Monte Carlo (ERP, *n* = 5)	71.83%	12.28
Monte Carlo (ERP, *n* = 7)	74.08%	13.39

14 EEG channels (ERP)	62.77%	7.47

ERP + spectral features	64.66%	5.79

Simulated online pipeline (ERP)	62.40%	4.80
